# The role of G-density in switch region repeats for immunoglobulin class switch recombination

**DOI:** 10.1093/nar/gku1100

**Published:** 2014-11-06

**Authors:** Zheng Z. Zhang, Nicholas R. Pannunzio, Chih-Lin Hsieh, Kefei Yu, Michael R. Lieber

**Affiliations:** 1 USC Norris Comprehensive Cancer Center, Molecular and Computational Biology Program, Departments of Biological Sciences; Pathology, Biochemistry & Molecular Biology; Molecular Microbiology & Immunology; Urology; University of Southern California Keck School of Medicine, 1441 Eastlake Ave., Rm. 5428, Los Angeles, CA 90089-9176, USA; 2 Department of Microbiology and Molecular Genetics, Michigan State University, 5175 Biomedical Physical Sciences, East Lansing, MI 48824, USA

## Abstract

The boundaries of R-loops are well-documented at immunoglobulin heavy chain loci in mammalian B cells. Within primary B cells or B cell lines, the upstream boundaries of R-loops typically begin early in the repetitive portion of the switch regions. Most R-loops terminate within the switch repetitive zone, but the remainder can extend a few hundred base pairs further, where G-density on the non-template DNA strand gradually drops to the genome average. Whether the G-density determines how far the R-loops extend is an important question. We previously studied the role of G-clusters in initiating R-loop formation, but we did not examine the role of G-density in permitting the elongation of the R-loop, after it had initiated. Here, we vary the G-density of different portions of the switch region in a murine B cell line. We find that both class switch recombination (CSR) and R-loop formation decrease significantly when the overall G-density is reduced from 46% to 29%. Short 50 bp insertions with low G-density within switch regions do not appear to affect either CSR or R-loop elongation, whereas a longer (150 bp) insertion impairs both. These results demonstrate that G-density is an important determinant of the length over which mammalian genomic R-loops extend.

## INTRODUCTION

The murine immunoglobulin heavy chain (IgH) locus consists of eight different constant (C) genes, most of which, except Cδ, contain a cytokine-inducible promoter and a highly repetitive switch (S) region (1,2). Class switch recombination (CSR) replaces the default Cμ exons with exons for a downstream constant chain (Cα, Cϵ or Cγ). Thus, instead of IgM, another isotype (IgA, IgE or IgG, respectively) is produced. During this process, two double-strand breaks, one in the donor Sμ and the other in the acceptor switch region, are created, and the intervening DNA region is deleted. Mammalian switch regions are all long, repetitive and G-rich on the non-template DNA strand, but there is no apparent homology among different switch regions, except for two short motifs (AGCT and GGGG) (3). These two motifs are enriched in mammalian switch regions relative to their neighboring regions, or elsewhere in the genome.

Transcription through switch regions is essential for CSR (3). The transcription of the IgH Sμ region is constitutive, but transcription of downstream regions is only active when certain cytokines are present at the time of antigen stimulation. A major role of transcription during CSR is to induce R-loop formation in the switch regions, which provides stable single-stranded DNA substrates for deamination of C to U by activation-induced deaminase (AID) (2,4,5). The G-richness on the non-template DNA strand is critical for R-loop formation in the switch region sequence in cell-free biochemical systems. The distance between the promoter and the switch regions reduces R-loop formation as well, probably due to a lower frequency of productive collisions between the G-rich portion of the transcript and the corresponding template DNA strand (6–8). Cellular (*in vivo*) studies at murine γ3 and γ2b regions have shown that most R-loops terminate within the switch regions, but some extend to within 600 bp downstream of the switch regions (9). The downstream boundaries of R-loops coincide with the zone where G-density gradually falls to the level of the genome average. However, no direct cellular analysis has been performed to evaluate the role of high G-density in mammalian switch regions during R-loop formation and CSR. Recently, we studied the role of G-clusters in initiating R-loop formation, but we did not evaluate the role of G-density in permitting the elongation of the R-loop once it had initiated (10).

In biochemical studies with purified DNA and prokaryotic RNA polymerases, G-clusters (2–4 directly adjacent G's) are important for the newly transcribed RNA to thread back onto the template DNA strand to form an R-loop (6–8). This is almost indistinguishable from the formation of R-loops at DNA replication origins in prokaryotes, where a G-cluster of a newly transcribed RNA must anneal to the template DNA strand to initiate DNA replication (11). Factors that favor annealing of the RNA to the DNA template, such as negative superhelicity, non-template DNA strand nicks and proximity of the G-cluster to the 5′ end of the RNA all favor more efficient R-loop formation. For switch sequences, we term the G-cluster rich region responsible for initiating the R-loops the ‘R-loop initiation zone’ or RIZ. Once initiated in the RIZ, we find that the R-loop can extend downstream of the RIZ into a region with no G-clusters but in which the G-density is relatively high (35–50% G). We call this region the ‘R-loop elongation zone’ or REZ.

Here, we vary the G-density in the elongation zone (REZ) of IgH switch regions in the genome of a murine B cell line, in contrast to our earlier genome study of G-clusters in the RIZ (10), to investigate its role in R-loop elongation. Consistent with our earlier *in vitro* studies (6–8), we find that a low G-density in the REZ dramatically decreases R-loop formation and CSR efficiency at the natural Sα locus in murine B cells. Short insertions (50 bp) within the switch regions do not appear to affect either CSR or R-loop elongation, regardless of the G-richness of the insertions. However, both CSR and R-loop elongation are impaired when longer, low G-density sequences are added within the switch regions. These findings explain the importance of a high G-density at mammalian switch regions.

## MATERIALS AND METHODS

### Cell culture and CSR assay

CH12F3.2a and its derivative cells were cultured in 10% fetal calf serum (FCS) RPMI medium supplemented with 50 μM β-mercaptoethanol. As for CSR assay, healthy cells in log phase were seeded at 5 × 10^4^ cells/ml in medium with or without cytokine stimulation (abbreviated CIT) as follows: 1 μg/ml anti-CD40 (eBioscience #16-0404-86), 5 ng/ml IL-4 (R&D #404-ML-010) and 0.5 ng/ml TGF- β1 (R&D #240-B-002), and grown for 72 h. Cells were stained with fluorescein isothiocyanate-conjugated anti-mouse IgA antibody (BD #559354) and analyzed by flow cytometry. CSR efficiency was determined based on the percentage of IgA+ cells minus the percentage without CIT stimulation.

### Plasmid construction

The 80 nt oligonucleotides corresponding to each REZ repeat unit were 5′-phosphorylated and purified on a denaturing polyacrylamide gel (Supplementary Table S1). A pair of complementary oligonucleotides was annealed and self-ligated at a final concentration of 4 μM. Ligation products were resolved on agarose gel, and DNA with expected length was recovered, blunted with Klenow and cloned into the exchange vector backbone. The 50-bp insertions were 5′-phosphorylated, annealed and cloned into the exchange vector backbone. The 150-bp insertion was amplified from a bacteria plasmid pGG49, and cloned into the exchange vector. The sequence of the exchange vectors was confirmed by DNA sequencing.

Given the DNA sequence complexity of IgH class switch regions, there are well over 10^100^ variations that one could consider constructing. Combined with our earlier study of G-clustering at the IgH switch regions (10), we have analyzed only slightly over 100 variations (each with 5–7 mammalian cell clones). Given the limited number of variations analyzed, we have tried to be cautious about extrapolating. Relevant to this, we do not endorse any of the current computer programs that predict R-loop formation based on DNA sequence (12).

### Cellular targeting and screening

Five micrograms exchange vector and one microgram Cre-expression vector were co-transfected into 1F7 cells by electroporation (Lonza). Transfected cells were serially diluted and seeded in 96-well plates. After 72 h, ganciclovir (Sigma-Aldrich #G2536-100MG) was added at a final concentration of 2 μg/ml. At 7 days after transfection, single clones were tested for puromycin sensitivity at a final drug concentration of 1 μg/ml. Puromycin-sensitive clones were screened by polymerase chain reaction (PCR) adjacent to the upstream and downstream boundaries of LoxP sites and across the entire switch region. A subset of clones were also examined by Southern blot. At least five clones were used for CSR assay.

### Native bisulfite sequencing

The principle of native (non-denaturing) bisulfite genomic sequencing is that all C's in duplex nucleic acid configuration (DNA:DNA or RNA:DNA) are resistant to bisulfite conversion from C to U (then remain to read as C during sequence analysis). But regions of single-stranded nucleic acid are subject to C to U (then read as T in the subsequent sequencing analysis) conversion (4,13).

For analyses, 1 million healthy cells at a density ∼10^5^ cells/ml were supplemented with anti-CD40, IL-4 and TGF-β1 for 48 h, and genomic DNA was extracted. Five micrograms of genomic DNA was incubated with the bisulfite solution (Lightning Conversion Reagent by Zymo Research D5030) for 16 h at 37°C, and recovered as described by the manufacturer. The switch regions were amplified with one converted primer and one native primer by PCR (Supplementary Table S2), and subcloned into the pGEM-T Easy vector (Promega A1360). At least 12 clones from each switch region were randomly chosen and analyzed.

As discussed in our earlier descriptions of bisulfite genomic sequencing on native nucleic acid, there is some level of background C to U (read as a T after PCR) even when there is no R-loop (4,13). Therefore, we set 80 bp as the minimal length of C conversion before we regard this as indicating an R-loop. In this study, each synthetic Sα repeat has 4 AGCT sites, and thus 4 C's. This means that four consecutive C conversions is the minimal length of an R-loop.

### S9.6 purification

ATCC HB-8730 hybridoma line (generously provided by Bradley Cairns) was cultured in a CELLine 1000 bioreactor (Satorius Biotech, NY, USA) according to manufacturer's instructions. Harvested antibody (culture supernatant) was purified on a column packed with Protein G Sepharose 4 Fast Flow (GE Healthcare) equilibrated with 1× phosphate buffered saline.

### S9.6 immunoprecipitation (IP)

Healthy cells in log phase were seeded at 3 × 10^5^ cells/ml in medium with anti-CD40, IL-4 and TGF-β1 and grown for 24 h. Genomic DNA was prepared by overnight proteinase K digestion, phenol-chloroform extraction and ethanol precipitation. Genomic DNA was digested with EcoRI in NEB buffer 2 [10 mM Tris-HCl, 10 mM MgCl2, 50 mM NaCl, 1 mM DTT pH 7.9]; importantly, RNase A, at a final concentration of 0.4 mg/ml, was added at this step to prevent S9.6 antibody binding to RNA species in subsequent steps (14). [In a separate methods study, we find that RNase A addition is needed to obtain adequate IP of RNA:DNA hybrids using S9.6. Without RNase A treatment, S9.6 yields a much lower (near background) IP (ZZZ, NP and MRL, submitted).] Five micrograms of fragmented genomic DNA was incubated with 5 μg S9.6 antibody in 400 μl IP buffer (10 mM sodium phosphate (pH 7.0), 140 mM NaCl, 0.1% Tween 20) for 2 h at 4°C. Ten microliter pre-blocked Dynabeads (Invitrogen 10004D) were added into the mixture and gently rotated at 4°C. After 2 h, beads were washed with IP buffer three times, and treated with proteinase K overnight. DNA bound to the beads was recovered by phenol-chloroform extraction, and quantified by quantitative (real-time) PCR (Supplementary Table S3).

### Germ-line transcript quantification

Two million healthy cells at a density of 10^6^ cells/ml were supplemented with anti-CD40, IL-4 and TGF- β1 for 6 h, and total RNA was extracted with GenElute^TM^ Mammalian Total RNA Kit (Sigma-Aldrich #RTN350). Note that 10% of RNA was reverse transcribed (RT) into cDNA with M-MuLV Reverse transcriptase (NEB M0253S), and 10% of the RT products were analyzed with quantitative PCR using Taqman probe/primers. β-Actin was used as an internal control. Each sample was done in duplicate, and at least three independent cellular clones were analyzed for each construct.

## RESULTS

### Experimental strategy

**Figure 1.**
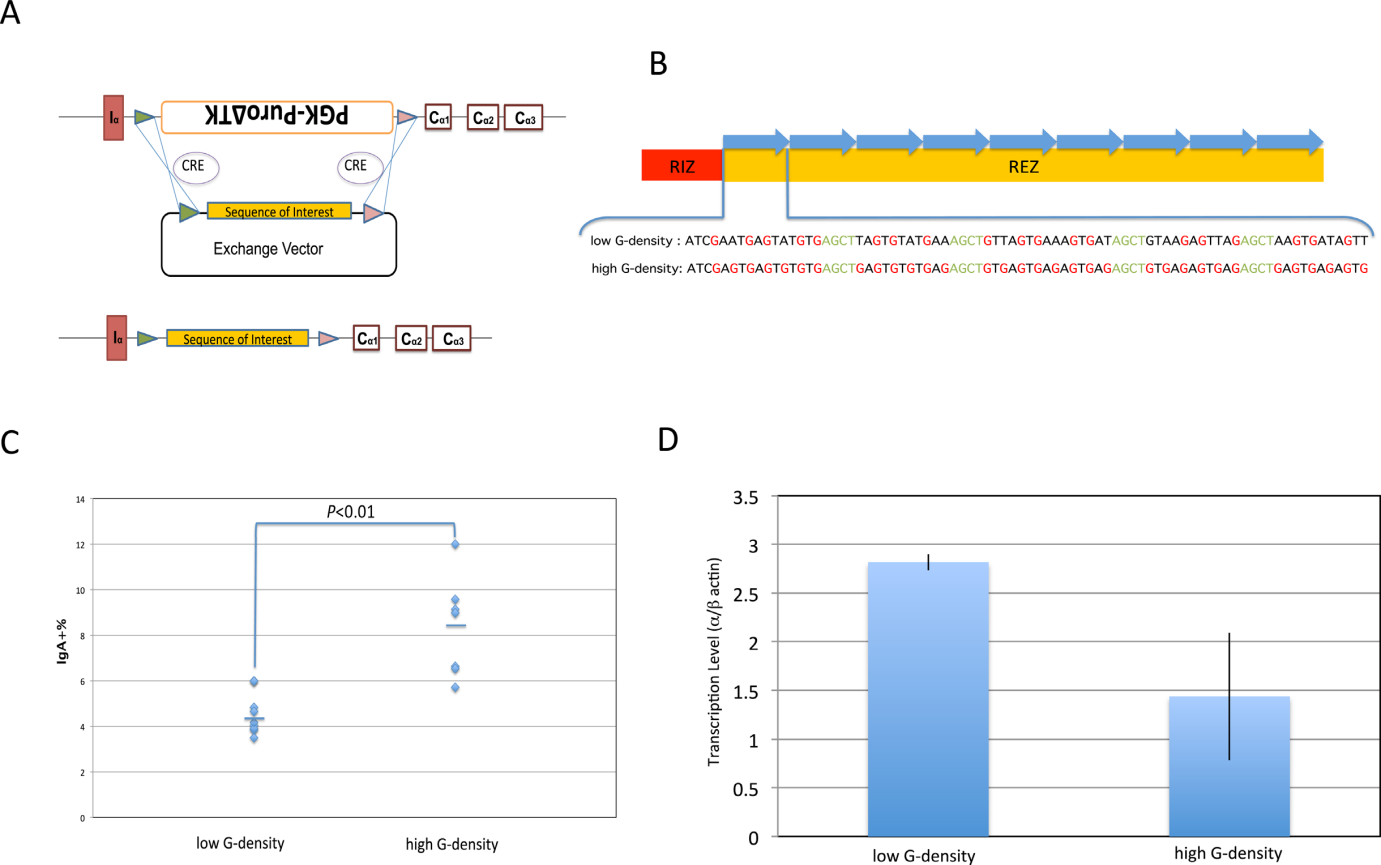
Overall G-density is important for efficient CSR. (A) 1F7 cells, which are resistant to puromycin and sensitive to ganciclovir, are transfected with both an exchange vector containing the sequence to be integrated (sequence of interest) and a CRE-expression vector. Cre mediates recombination between the exchange vector and the 1F7 chromosome at the corresponding LoxP sites. Cells that have successfully replaced the selection cassette by the sequence of interest would survive during ganciclovir selection, which are further screened with a puromycin sensitivity test and PCR. (B) Nine repeats of 80 bp oligonucleotides (top strand of duplex shown below the RIZ/REZ diagram) with 4 AGCT sites (orange) and different G-densities were used for the REZ in the synthetic switch regions. The top panel has 29% G-density on the non-template DNA strand, whereas the bottom panel has 46% G-density. The RIZ contains 5 G-clusters. (C) Fluorescence-activated cell sorting (FACS) analysis of CSR is shown. Each data symbol represents an independent clone (after subtraction of the IgA+ level for the same clone without CIT stimulation). The bars represent the mean of each group. (D) Germ-line transcription (GLT) Quantification. Healthy cells grown to 10^6^/ml were treated with 1 μg/ml anti-CD40, 5 ng/ml IL-4 and 0.5 ng/ml TGF-β1 (CIT) for 6 h. Total RNA was extracted, reverse-transcribed into cDNA and quantified by real-time PCR. β-Actin was used as an internal control. In parallel, the same cellular clones without CIT treatment were used to measure the background of GLT. The error bar represents the SEM of 3–5 independent clones.

To examine the importance of a high G-density in mammalian switch regions, we used a system that allows us to exchange out the genomic switch region and replace it with any desired synthetic switch sequence in a mouse B-cell line, CH12F3.2a (15). Such an exchange permits us to study how switch sequence variations affect R-loop formation and cellular CSR *in vivo*. This cell line is able to specifically and efficiently switch to IgA upon cytokine stimulation. Using this cellular assay system, the endogenous Sα locus in CH12F3.2a was replaced with a positive-negative selection cassette (PuroΔTK, provides puromycin resistance and ganciclovir sensitivity) flanked by two different LoxP sites (10,15). The resulting cell line is called 1F7. The sequence of interest is cloned into an exchange vector with the same LoxP sites as 1F7 cells. This exchange vector is cotransfected into 1F7 cells along with a Cre-expressing vector. Cre mediates recombination between the exchange vector and the 1F7 chromosome at the corresponding LoxP sites (Figure 1A). The successful replacement of the selection cassette by the sequence of interest gives rise to ganciclovir resistant clones, which are screened with a puromycin sensitivity test and PCR analysis for all clones. The CSR assay is described in the Materials and Methods. R-loop formation is determined using native bisulfite genomic sequencing and by IP using an RNA:DNA antibody called S9.6 (see Materials and Methods).

### Overall G-density is important for efficient CSR

Mammalian switch regions are extremely G-rich on the non-template DNA strand. G-clusters have been shown to be critical for CSR (10). Previous *in vitro* experiments indicate that a high G-density is important for CSR, but no direct *in vivo* evidence has addressed this issue (9,16).

To evaluate the potential importance of high G-density for CSR, we constructed two cell lines with exactly the same length (9 repeats) and number of AGCT sites (4 within each repeat), but different G-densities on the non-template DNA strand (Figure 1B). One has an average G-density of 29% on the non-template DNA strand, whereas the other one has an average G-density of 46%. Using the same RIZ, we find that an REZ with higher G-density causes a significant increase in CSR (Figure 1C). The high G-density REZ leads to a decrease in transcription compared with a low G-density REZ (Figure 1D). The drop in transcription may be due to obstruction of the RNA polymerase by the R-loop, as we have seen in our biochemical studies (17). Regardless of the cause, this finding rules out increased transcription as the cause of the increased CSR, and clearly indicates that higher G-density in the REZ results in higher CSR.

### High G-density is important to maintain R-loops

Given the identical RIZ and number of AGCT sites, the R-loop initiation and AID target number (WGCW site number) are both the same between the two constructs above. The only difference, G-density within the REZ, may affect R-loop elongation, based on our earlier cell-free biochemical observations (6–8).

**Figure 2.**
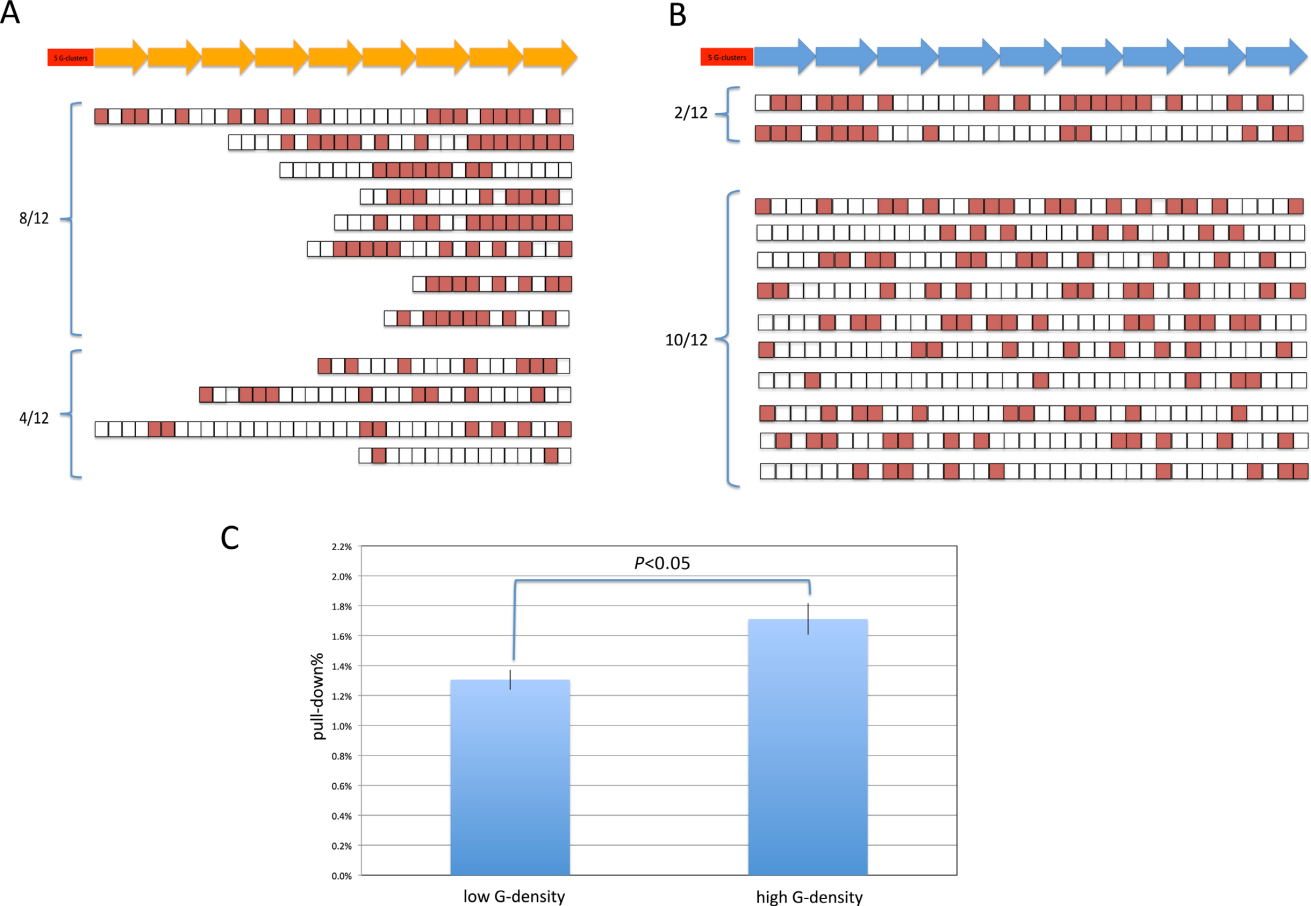
Longer and more frequent R-loops are detected using bisulfite sequencing when the REZ has a high G-density. Bisulfite sequencing analysis was done on the cellular constructs. Detailed R-loop information is shown with high (A) or low (B) overall G-density on the non-template DNA strand, respectively. Solid boxes represent C nucleotides that have been converted to U (and then T) nucleotides due to bisulfite. The results are grouped according to alleles that contain at least the equivalent of one 80 bp repeat unit of C conversion. This corresponds to four or more consecutive C conversions (top). Those with fewer than four consecutive C conversions (less than one 80 bp repeat unit of conversion) are shown in the lower group and designated as short regions of conversion. (C) IP with the S9.6 antibody was performed on the cellular constructs with different overall G-density in Figure 1. Background signals from mock samples with no antibody were subtracted. Values were normalized to the total input DNA to calculate the pull-down percentage. Three independent IP experiments were performed for each cell line.

We wondered if R-loops collapse more in the switch region with a low G-density on the non-template DNA strand. We performed bisulfite sequencing for the cellular constructs above. We find that 8 out of 12 of the molecules have long stretches of conversion when the G-density is high in the REZ (see legend to Figure 2A), and only 2 out of 12 when the G-density is low in the REZ (Figure 2B). Moreover, the ratio of converted C nucleotides in the cell line with the higher G-density on the non-template DNA strand is higher than that with the lower G-density (44% versus 31%, *P* < 0.05). No converted C nucleotides were observed on the template DNA strand.

To further evaluate the relationship between G-density and R-loop formation, we used an antibody, called S9.6, to detect R-loops (Figure 2C). This antibody has been documented to preferably bind RNA:DNA hybirds over DNA:DNA or RNA:RNA duplexes, if free RNA is removed using RNase A. We find that there is consistently more pull-down of R-loops at this location of the genome when an REZ with 46% G (non-template strand) is used rather than 29%.

The difference in S9.6 pulldown is not large, though it is quite reproducible (Figure 2C), likely because the short R-loops that are expected to arise in the RIZ are sufficient to permit S9.6 pull-down.

Therefore, a high G-density in the REZ supports more frequent R-loop elongation within the cells.

### R-loops can proceed across a short region with low G-density

Although the distance of DNA between switch regions and their upstream promoters (typically 200–600 bp in length) are not G-rich (on the non-template strand) at mammalian IgH loci, a few G-clusters (e.g. three G-clusters of 3 or 4 nt each at Sα) typically exist within these regions. The R-loops initiated from those G-clusters may extend into the switch regions, where the R-loop extension may procede efficiently. Thus, we wondered how regions of low G-density may affect R-loop stability between regions of high G-density.

**Figure 3.**
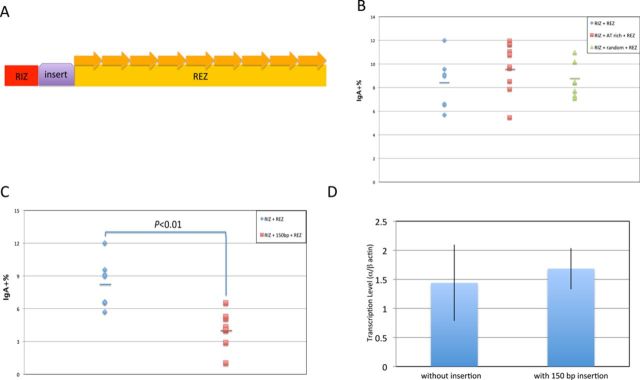
R-loops can proceed across a short region of DNA that has a low G-density. (A) DNA with different lengths (purple) were inserted between RIZ (containing 5 G-clusters) and REZ (containing 9 repeats with high G-density and 4 AGCT sites within each repeat). (B) A 50-bp segment of DNA with either AT-rich sequence or random sequence was inserted between RIZ and REZ. FACS analysis is shown. Each data symbol represents an independent clone. The bars represent the mean of each group. (C) A 150-bp segment of DNA with AT-rich sequence was inserted between RIZ and REZ. FACS analysis is shown. Each data symbol represents an independent clone. The bars represent the mean of each group. The Student's *t*-test was used to analyze the effect of the insert. (D) Transcription assay for the clones in panel C. The same method was used as described in Figure 1D and in the Materials and Methods. We observe no significant difference in transcription here in panel D.

To explore whether R-loops can tolerate short regions of low G-density, we inserted two pieces of DNA with different sequences between the RIZ and the REZ (Figure 3A). If R-loops are sensitive to G-density and collapse once the G-density is low, then the R-loops that begin in the RIZ would not reach the REZ portion, and AID/UNG/APE1 would not have an opportunity to create DNA breaks. In this case, CSR efficiency would drop dramatically.

We find that CSR efficiency is not affected by the 50-bp insertion between the RIZ and REZ (Figure 3B). However, when the size of the insert between the RIZ and REZ is increased to 150 bp with a low G-density sequence on the non-template DNA strand, the CSR does drop substantially (Figure 3C), even though transcription is unaffected (Figure 3D).

To evaluate whether there is a correlation between the CSR efficiency and the R-loop status, we performed bisulfite sequencing for the cellular clones above. We found that with the short insertion (50 bp), regardless of the G-richness, R-loop status is not affected (Table 1), which agrees with the fact that CSR efficiency of these cellular constructs is not affected. However, fewer R-loops are observed with a longer (150 bp) insertion of low G-density (Table 1). We conclude that R-loops can tolerate short interruptions by a sequence of low G-density.

**Table 1.** R-loop analysis using bisulfite sequencing on different cellular constructs

		Mean% IgA^+^	% R-loop (actual #)
1	RIZ + REZ	8.4	67 (8/12)
2	RIZ + 50bp AT-rich + REZ	9.8	73 (16/22)
3	RIZ + 50bp random + REZ	8.6	60 (12/20)
4	RIZ + 150bp AT-rich + REZ	4.2	31 (11/36)

**Figure 4.**
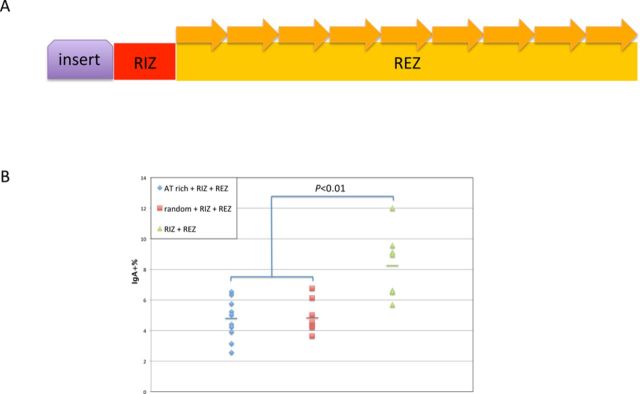
Distance between G-clusters and promoter influences CSR. (A) The same 50 bp DNA segment as in Figure 3B was inserted immediately upstream of the RIZ (the RIZ contains 5 G-clusters). (B) FACS analysis is shown. Each data symbol represents an independent cellular clone. The bars represent the mean of each group. The Student's *t*-test was used to analyze the effect of the insert.

We also tested whether insertion of 50 bp upstream of the entire RIZ/REZ region would affect CSR within cells (Figure 4A). Our cell-free biochemical work showed that a greater distance between the transcriptional promoter and the RIZ/REZ region reduces R-loop formation (8). Consistent with our cell-free biochemical studies, CSR is reduced when the distance between the promoter and the RIZ/REZ switch sequences is increased (Figure 4B).

## DISCUSSION

Based on the much higher ratio of [switched isotype (e.g. IgG in mammals or IgX in amphibians) divided by unswitched isotype (IgM)] in mammals versus more distantly evolved vertebrates, we have speculated that mammalian Ig CSR may be more efficient than other species that carry out CSR. If so, some of the greater efficiency may be due to the distinctively G-rich nature of the switch sequence on the non-template strand. Upon transcription through switch regions, G-richness on the non-template DNA strand favors R-loop formation. R-loop formation provides stable single-stranded DNA, which is required as a substrate for AID. The *in vivo* boundaries of R-loops have been documented (9,16), and G-clusters have been demonstrated to be crucial for R-loop initiation *in vitro* and *in vivo* (6–8,10). Decline in G-density on the non-template DNA strand is thought to account for the collapse of R-loops, but no direct evidence has been reported. Here, we have shown that decreasing G-density at mammalian IgH switch regions impairs both R-loop formation and CSR (Figures 1 and 2).

We find that the distance between switch regions and their respective promoters affects CSR efficiency (Figure 4). This agrees with the biochemical studies on R-loop formation (8). Upstream portions of the RNA transcript that are not G-rich may reduce annealing of the G-rich portion of the RNA to the C-rich template DNA. Mammalian switch regions are located several hundred base pairs downstream of their respective promoters. Though these sequences between switch regions and promoters are not G-rich, ample G-clusters are found within these regions. Our finding that 50 bp insertions between RIZ and REZ do not affect CSR indicates that R-loops can extend across short regions with low G-density. In this case, R-loops initiated from the G-clusters upstream of switch regions may extend and reach switch regions, or they may increase the frequency of collisions between RNA and DNA around switch regions, where longer and more stable R-loops can form.

The length of low G-density sequence through which R-loops can persist likely accounts for the wide spectrum of downstream boundaries of R-loops at mammalian switch regions. The downstream boundaries do not have a distinct point of R-loop termination at the end of the zone of high G-density (9). Rather, they are distributed up to 600 bp downstream, as the G-density gradually declines below 35%. The persistence of R-loops through regions of low G-density (50 bp here) illustrates that R-loops do not terminate acutely at a boundary of high to low G-density.

Overall, we see very good correlation between our cell-free biochemical studies of R-loop formation (6–8) and *in vivo* cellular R-loop formation and CSR at the mammalian chromosomal level. The study here focused on the G-density in the REZ. In our earlier study of how G-clusters affect CSR *in vivo*, we included some constructs where G-clusters were embedded in the REZ repeats (10). The results from those constructs were consistent with the findings here in indicating that higher G-density in the REZ increases CSR. The results here are more directly interpretable because only the G-density is varied, and not the G-clustering.

We have not done a formal test of how R-loops interfere with RNA polymerase II progression. We do note that the total number of transcripts produced *in vitro* when purified prokaryotic RNA polymerases are used to transcribe through switch sequences on purified DNA templates is lower than if random sequences are used (17). Perhaps RNA polymerase moves more slowly through GC-rich sequences. It is known that RNA polymerase II accumulates in switch regions (18,19). It is possible that such accumulation of RNA polymerase contributes to the stability of R-loops once formed.

There have been numerous recent reports of R-loops at other locations in the eukaryotic genome (14,20–32), outside of the IgH locus. Nearly all of these studies rely heavily on the S9.6 antibody for immunostaining or IP of RNA:DNA duplexes. As mentioned in the Materials and Methods, we find important differences in the IP of the IgH switch regions, depending on whether the harvested genomic nucleic acid is treated with RNase A prior to IP (ZZ, NP and MRL, submitted). In light of our experience and in light of studies demonstrating S9.6 binding to RNA:RNA (14,33), it may be useful to confirm the existence of R-loops at other genomic locations using the same types of functional and biochemical studies that have been applied to the IgH switch regions over the last 25 years (2–8,13,17,34–38). If these other locations are confirmed, then the rules for R-loop formation defined here will help understand the broader contribution of R-loops to physiologic and pathologic events in the nucleus.

## SUPPLEMENTARY DATA

Supplementary Data are available at NAR Online.

SUPPLEMENTARY DATA
