# Supplementary material for: The role of G-density in switch region repeats for immunoglobulin class switch recombination
Source: Nucleic Acids Res. 2014 Nov 6;42(21):13186–93. doi: 10.1093/nar/gku1100 (PMC4245955; doi:10.1093/nar/gku1100)
Supplement: SUPPLEMENTARY DATA [file supp_42_21_13186__index.html]

The role of G-density in switch region repeats for immunoglobulin class switch recombination — The role of G-density in switch region repeats for immunoglobulin class switch recombination — SUPPLEMENTARY DATA 

# The role of G-density in switch region repeats for immunoglobulin class switch recombination

## SUPPLEMENTARY DATA

**Files in this Data Supplement:**

- SUPPLEMENTARY DATA
