# Supplementary material for: The role of G-density in switch region repeats for immunoglobulin class switch recombination
Source: Nucleic Acids Res. 2014 Nov 6;42(21):13186–93. doi: 10.1093/nar/gku1100 (PMC4245955; doi:10.1093/nar/gku1100)
Supplement: SUPPLEMENTARY DATA [file supp_gku1100_nar-02858-v-2014-File003.pdf]

Table S1. Construction Oligonucleotides and Sequences.

	Sequence	Function
1	TGGGGTCGGGCCTCGAGGGGAATTCTAGGGTACCATGGGA	RIZ with 5 G-clusters
2	GATCTCCCATGGTACCCTAGAATTCCCCTCGAGGCCCGACCCCATGCA	complementary to 1
3	ATCGAGTGAGTGTGTGAGCTGAGTGTGTGAGAGCTGTGAGTGAGAGTGA GAGCTGTGAGAGTGAGAGCTGAGTGAGAGTG	one REZ repeat unit with 4 AGCT sites (high G-density)
4	CTCGATCACTCTCACTCAGCTCTCACTCTCACAGCTCTCACTCTCACTCA CAGCTCTCACACACTCAGCTCACACACTCA	complementary to 3
5	ATCGAATGAGTATGTGAGCTTAGTGTATGAAAGCTGTTAGTGAAAGTGATA GCTGTAAGAGTTAGAGCTAAGTGATAGTT	one REZ repeat unit with 4 AGCT sites (low G-density)
6	TTCGATAACTATCACTTAGCTCTAACTCTTACAGCTATCACTTTCACTAACA GCTTTCATACACTAAGCTCACATACTCA	complementary to 5
7	GATCTTATAGAATATTATGTATTTAAAGTTCATAGTATTTCAGATCTATTATGTA G	insert 50bp AT-rich sequence between the RIZ and the REZ
8	TCGACTACATAATAGATCTGAAATACTATGAACTTTAAATACATAATATTCTAT AA	complementary to 7
9	GATCTTTCGAAGTCGTCATGTCAGTCTGAGAAGTTCAGTCGAGTCGTCAG ATCGTG	insert 50bp random sequence between the RIZ and the REZ
10	TCGACACGATCTGACGACTCGACTGAACTTCTCAGACTGACATGACGACT TCGAAA	complementary to 9
11	GGCAGGAGATCTAACTCTTTGTCCAATCTCTCTTTC	PCR primer to amplify the 150 bp insertion
12	GCCACTAGTGTCGACAGTGAGTTTTTGTTAGAAAAGAGAA	Paired with 11
13	TATGTATAGAATATTATGTATTTAAAGTTCATAGTATTTCAGATCTATTATGTAA	insert 50bp AT-rich sequence upstream of the RIZ
14	CTAGTTACATAATAGATCTGAAATACTATGAACTTTAAATACATAATATTCTAT ACA	complementary to 13
15	TATGTTCGAAGTCGTCATGTCAGTCTGAGAAGTTCAGTCGAGTCGTCAGA TCGTA	insert 50bp random sequence upstream of the RIZ
16	CTAGTACGATCTGACGACTCGACTGAACTTCTCAGACTGACATGACGACT TCGAACA	complementary to 15
17	AGATCTAACTCTTTGTCCAATCTCTCTTTCCACTCCACAATTCTGCTCTGAA TACTTTGAGCAAACTCAGCCACAGGTCTGTACCAAATTAACATAAGAAGCA AAGCAATGCCACTTTGAATTATTCTCTTTTCTAACAAAAACTCACTGTCGAC	PCR product of 11 and 12

Table S2. PCR Primers for Diagnosis and Bisulfite Sequencing Oligonucleotides.

	Sequence	Function
ZZ46	TCACCCAGTCCTAAGCTCTCTACC	forward primer in the Iα
ZZ49	GTAGTCTTCACACTGCCCATCTCG	reverse primer near the first Cα
ZZ103	CACTCGATATCGTCGACAGATCTCC	diagnose the insertion of DNA with (5/6 or 7/8 +)1/2 + 3/4, paired with ZZ46
ZZ104	GTGATCGAGATCGGATCCATCGAT	diagnose the insertion of DNA with (5/6 or 7/8 +)1/2 + (9/10 or 11/12 +) 3/4, paired with ZZ49
ZZ463	GATCTCCCATGGTACCCTAGAATTCC	diagnose the insertion of DNA with 1/2 + 9/10 or 11/12 + 3/4, paired with ZZ46
ZZ455	GTCGACAGATCTCCCATGGTACC	diagnose the insertion of DNA with 1/2 + 13/14, paired with ZZ46
ZZ457	AGCTAAGTGATAGTTAATCGGATCCATCG	diagnose the insertion of DNA with 1/2 + 13/14, paired with ZZ49
ZZ465	GGGTTTTGAGGGGAATTTTAGGGTATT	forward converted primer for bisulfite sequencing
ZZ471	CTCGATCTAGAGAACCCCTTCATCG	reverse native primer for bisulfite sequencing

Table S3. qPCR Primers and Probes

	Sequence	Function
S9.6 ChIP		
ZZ304	[6-FAM]CCCAGTGATAATCGGCTGCCTGATTC[BHQ1a-6FAM]	probe for the Sα region
ZZ305	CCCACGAGCTCTGTCAAGTG	forward primer for the Sα region
ZZ306	GCCGGAAGGGAAGTAATCG	reverse primer for the Sα region
GLT Quantification		
ZZ274	CCTATGAAGGACACTCAACAACATTG	forward for the α region
ZZ275	CCGATTATCACTGGGTCACTTG	reverse primer for the α region
ZZ276	[6-FAM]ATCTACCCACTGACACTCCCACGAGCTC[BHQ1a-6FAM]	probe for the α region
ZZ277	TGCTCCCCGGGCTGTATT	forward primer for β-actin region
ZZ278	ACATAGGAGTCCTTCTGACCCATT	reverse primer for β-actin region
ZZ279	[6-FAM]ATCGTGGGCCGCCCTAGGCAC[BHQ1a-6FAM]	probe for the β-actin region
